# Two New Oxodolastane Diterpenes from the Jamaican Macroalga *Canistrocarpus cervicornis*

**DOI:** 10.3390/md15060150

**Published:** 2017-05-30

**Authors:** Sanjay Campbell, JeAnn Murray, Rupika Delgoda, Winklet Gallimore

**Affiliations:** 1 Department of Chemistry, University of the West Indies, Mona Campus, St. Andrew JMAAW15, Jamaica; sanjay.campbell@mymona.uwi.edu; 2 Natural Products Institute, University of the West Indies, Mona Campus, St. Andrew JMAAW15, Jamaica; jeann.murray02@uwimona.edu.jm (J.M.); thejani.delgoda@uwimona.edu.jm (R.D.)

**Keywords:** dolastane, diterpenes, *Canistrocarpus cervicornis*

## Abstract

The chemical investigation of the organic extract of *Canistrocarpus cervicornis,* collected at Drunken Man’s Cay at Port Royal, Jamaica, has led to the isolation of two new dolastane diterpenes 4*R*-acetoxy-8*S*,9*S*-epoxy-14*S*-hydroxy-7-oxodolastane (**1**) and 4*R*-hydroxy-8*S*,9*S*-epoxy-14*S*-hydroxy-7-oxodolastane (**2**) and the previously isolated dolastane (4*R*,9*S*,14*S*)-4,9,14-trihydroxydolast-1(15),7-diene (**3**) as a major diterpene constituent. The structures of the new compounds were elucidated by extensive spectroscopic analyses. Compounds **1**–**3** were evaluated for their cytotoxicity against human tumor cell lines PC3 and HT29. The results revealed that the dolastane diterpenes (**1**–**3**) displayed moderate, concentration dependent, cytotoxicity.

## 1. Introduction

Plant-derived drugs remain an important resource, especially in developing countries, and marine organisms provide a rich source of nutraceuticals and are potential candidates for the treatment of several human diseases [1]. Cancer is a leading cause of deaths worldwide, accounting for 8.8 million deaths in 2015 [2]. The most common causes of cancer deaths are cancers of the lungs, liver, colon, stomach, and breast.

Over the years, brown algae have caught the attention of many scientists in search of diverse and novel chemical structures. Marine algae of the order Dictyotales, (Phaeophyta) have been found to be distributed in tropical and sub-tropical waters. Chemical investigations have shown the order to be a rich source of monocyclic, bicyclic and tricyclic terpenes such as dolabellane, meroditerpenoid, dolastane and seco-dolastane skeleton types [3,4,5,6,7,8,9,10,11,12,13,14]. Quite a number of diterpenes belonging to the Dictyotaceae family, inhabitants of the Atlantic and Indian Oceans, belong to the dolastane and seco-dolastane types. The species *Canistrocarpus cervicornis* (formerly classified as *Dictyota cervicornis*) has been shown to grow luxuriantly in Caribbean waters and are commonly found [3].

**Figure 1:**
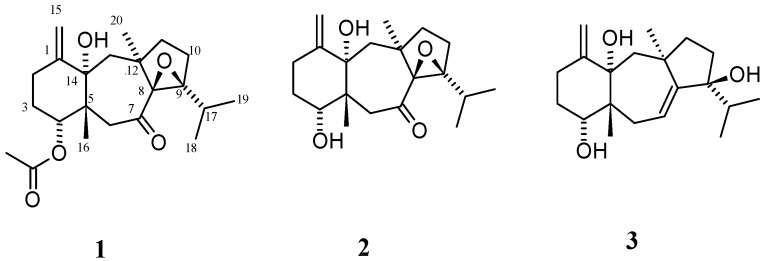
Chemical structures of compounds **1**–**3** isolated from *Canistrocarpus cervicornis*.

The chemical investigation of this macroalga has led to the isolation of a variety of dolastanes and seco-dolastanes with marked biological/ecological activity [4,5,6,7,9]. They display a wide variety of pharmacological activities including antifouling, antifeedant, antibacterial, antiviral, and cytotoxic properties [4,5,6,7,9]. In the course of our ongoing efforts to discover new biologically active secondary metabolites from brown algae, *Canistrocarpus cervicornis*, collected from off the coast of Port Royal, Drunken Man’s Cay, Jamaica, was investigated. We report herein that the CH_2_Cl_2_ extract of the dried brown alga resulted in the isolation and structure elucidation of two new oxodolastanes (**1** and **2**) and one known dolastane (**3**). The structure of the known dolastane (**3**) was identified by comparison of its physical and spectroscopic data with those reported in the literature (Figure 1). In addition, the cytotoxicity of the isolated compounds against human tumor cell lines PC3 and HT29 is also described.

## 2. Results and Discussion

A series of chromatographic separations of the CH_2_Cl_2_ extracts of the brown alga *Canistrocarpus cervicornis*, collected at Drunken Man’s Cay in Port Royal, Jamaica, resulted in the isolation of the new dolastanes **1** and **2** (Figure 1) and one previously reported dolastane, which was identified as (4*R*,9*S*,14*S*)-4,9,14-trihydroxydolast-1(15),7-diene (**3**) by comparison of its spectroscopic and physical characteristics with those reported in the literature [8].

### 2.1. Structural Elucidation

Compound **1** was isolated as a colorless gum. HRESIMS in positive ion mode showed a [M + Na]^+^ peak at *m*/*z* 399.2029 implying the molecular formula of C_22_H_32_O_5_ (seven degrees of unsaturation) for compound **1**. One olefinic carbon-carbon double bond and the two carbonyl groups accounted for three of the seven degrees of unsaturation, therefore the molecular structure of **1** was determined to be tetracyclic. The ^13^C-NMR spectral data revealed the presence of 22 carbon resonances, which were assigned by distortionless enhancement by polarization transfer (DEPT) and heteronuclear single quantum coherence (HSQC) spectra to eight non-protonated carbons, seven methylenes, five methyls, and two methines. A detailed analysis of 1D and 2D NMR spectra was necessary to determine the gross structure of **1**. The evidence of an acetate residue could be easily deduced from the ester carbonyl signals at δ_C_ 169.0 along with the methyl group at δ_H_ 2.15. Therefore, the diterpene structure of **1** was inferred from the remaining 20 carbons. NMR signals also revealed the presence of an oxygenated methine at δ_C_ 81.7 which was attached to the proton signal at δ_H_ 4.81 (1H, br s) which was assigned to an acetoxy-bearing methine.

An indication of the existence of an exocyclic double bond was ascertained from the methylene carbon NMR signals observed at δ_C_ 110.7 with olefinic methylene protons at δ_H_ 5.01 (1H, br s) and 4.98 (1H, br s), along with an olefinic quaternary carbon at δ_C_ 149.6. Furthermore, the existence of a ketone functionality was inferred from the carbon signal at δ_C_ 207.7. The quaternary signals at δ_C_ 82.0, 78.2, and 72.0 were characteristic of oxygen-bearing quaternary carbons, two of which were deduced to be oxirane carbon atoms at 82.0 ppm (C-9) and 72.0 ppm (C-8).

**Figure 2:**
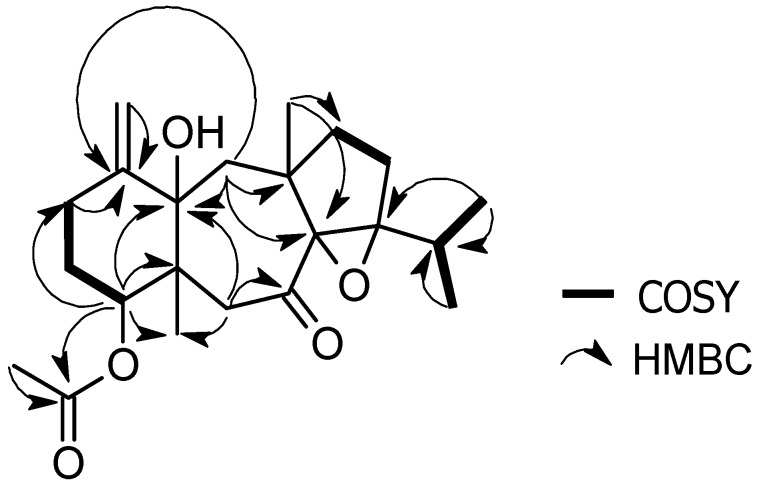
Key COSY and HMBC correlations observed for **1**.

Analysis of the presented NMR data revealed a close alignment to the tricyclic dolastane skeleton, which appears to be a skeletal framework characteristic to this alga species [1,2,3,4,5,6,7,8,9,10,11]. However, differences in the chemical shifts because of the oxygenation pattern suggested that compound **1** was a new natural product. ^1^H-^1^H correlation spectroscopy (COSY) experiments established the fragments outlined in Figure 2 after assignments of the direct ^1^H-^13^C correlations via HSQC analysis.

^1^H-^1^H COSY correlations between the methine proton at δ_H_ 2.74 (H-17) and the methyls at δ_H_ 0.95 (Me-18) and δ_H_ 0.96 (Me-19), suggested the occurrence of a terminal isopropyl fragment in the structure. Other important correlations were observed between olefinic methylene protons at δ_H_ 5.01 and 4.98 (H-15) with δ_H_ 2.66 (H-2), methylene protons at δ_H_ 1.92 and 1.83 (H-3) with the acetoxy-bearing methine at δ_H_ 4.81 (H-4), and methylene protons at δ_H_ 1.83 (H2-10) with 1.31 (H2-11). (Table 1, Figure 2).

Further structural information for compound **1** was obtained by 2D analysis; heteronuclear multiple bond correlation (HMBC) spectra (Figure 2). The location of the acetate group at C-4 was established through the HMBC correlations between δ_H_ 4.81 proton (H-4) and the carbonyl signal at δ_C_ 169.0. HMBC correlations of C-1, C-14, and C-15 to H-2, located the exocyclic double bond as a part of the six-membered ring and placed the hydroxyl group at C-14. Also, HMBC correlations of C-5, C-7, C-14, to H-6 and C-8, C-12, C-14, with H-13 established the position of the ketone functionality and allowed for the assignment of the seven-membered ring. Similarly, the epoxy C-atom, C-9, was HMBC correlated to H-17, H-18, and H-19 confirming the ether bridge and the isopropyl fragment on the five-membered ring. Based on the above evidence, the planar structure of dolastane **1** could be established as 4-acetoxy-8,9-epoxy-14-hydroxy-7-oxodolastane.

**Figure 3:**
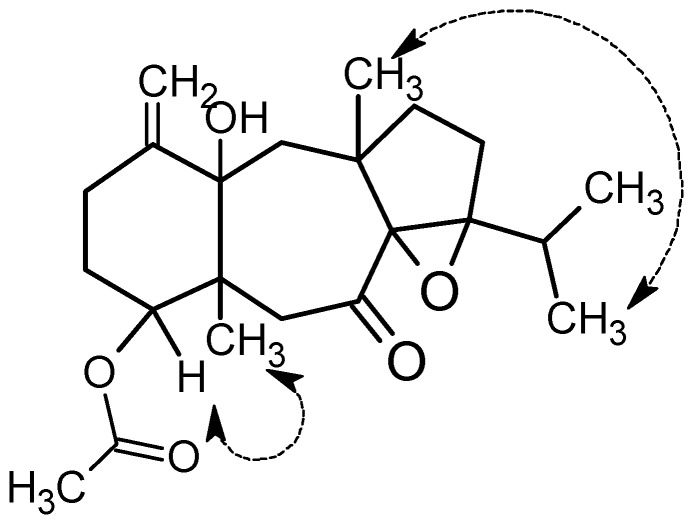
Key NOESY correlations for **1**.

The relative configuration of compound **1** was established by extensive analysis of nuclear Overhauser effect spectroscopy (NOESY) spectrum. The assignment of the stereocenter was based on careful analysis of the NOESY spectrum as well as comparison with the reference [8]. Additionally, OH-Me ^1^H NMR shifts effect helped with resolving the relative configuration. It is of note that, when an H or Me group is coplanar to a nearby OH group, it is deshielded. The usual mean position of a methyl substituent in a 1,3 relationship with a hydroxyl group is 0.72 ppm. Consequently, the 14*S*-hydroxy stereocenter was deduced from the ^1^H-NMR signal of Me-20 at 1.37 ppm, deshielded by 0.65 ppm from the mean, thus co-facial with H_3_-20 [15,16]. In addition, strong NOE interaction between H-4 and H_3_-16 provided evidence that they were co-facial (Figure 3). This inferred *trans*-fusion of the six and seven membered ring. The absence of nuclear Overhauser effect (NOE) between H_3_-16 and H_3_-18 suggested that they were on opposite sides, while the NOE enhancement of H_3_-20 ad H_3_-18 (Figure 3) provided evidence for the *trans*-fusion of the seven and five membered rings and determining the relative configuration of the chiral centers C-4, C-5, C-8, C-9, C-12, C-14, as 4R*,5R*,8S*,9S*,12R*, and 14S*.

Compound **2**, obtained as a white solid, gave the molecular formula C_20_H_30_O_4_, as calculated from the [M + Na]^+^ pseudo molecular ion at *m*/*z* 357.1923 and NMR data. The spectroscopic characteristics of **2** were comparable to those of compound **1**. Thus, the gross structure of **2** was determined by comparison of the spectroscopic data with that of compound **1**. By contrast, the differences between **1** and **2** lie in the NMR signal at δ_H_ 3.53 connected to δ_C_ 78.7, assigned as a hydroxy methine at C-4 due to its upfield shift with respect to δ_H_ 4.81 connected to carbon at δ_C_ 81.7, in compound **1** and the absence of the chemical shifts that correspond to the acetate group in compound **1**. Furthermore, HMBC correlations established the hydroxyl group at C-4 methine as H-4 correlated with C-1, C-2, C-5, C-14, and C-16 in the HMBC spectrum. Based on the above evidence, the planar structure of dolastane **2** could be established as 4-hydroxy-8,9-epoxy-14-hydroxy-7-oxodolastane.

The NOE interactions of H_3_-20/H_3_-18 and H-4/H_3_-16 suggested the same relative configurations at C-4, C-5, C-8, C-9, C-12, and C-14 as in the case of **1**.

It should be noted that the occurrence of the epoxide in the dolastanes remains uncommon with only one other such isolate being identified. Spectroscopic data and X-ray diffraction analysis provided evidence for this isolate to be identified as 10β-acetoxy-8α,9α-epoxy-14β-hydroxy-7-oxodolastane [1].

Spectral data (^1^H, ^13^C NMR, MS, optical rotation) of compound **3** (53 mg) are the same as those of (4*R*,9*S*,14*S*)-4,9,14-trihydroxydolast-1(15),7-diene which was previously isolated from *Canistrocarpus cervicornis*. This compound has been described with noteworthy anti HSV-1 and anti-HIV-1-RT activity [7].

### 2.2. Cytotoxicity Evaluation against PC3 and HT29 Cancer Cell Lines

Evaluation of the impact of compound **1** on the viability of the human prostate adenocarcinoma cell line—PC3—and the human colon adenocarcinoma cell line—HT29—revealed moderate, concentration-dependent cytotoxicity. The EC_50_ value, the concentration at which cell viability is decreased by 50%, was estimated to be above 100 µM for both PC3 and HT29. Sixty-eight percent (68%) and 59% cell viability was observed respectively for these two cell lines at 133 µM of compound **1**. Such patterns are comparable with the chosen positive control drug, bicalcutamide for PC3 cells which yielded an EC_50_ value of 103.9 µM. Bicalcutamide is known to have no effect on PC3 at low doses, but significant cytotoxicity against PC3 at high doses [17,18,19,20]. More potent activity was observed for positive control drugs, doxorubicin and tamoxifen, against HT29 with EC_50_ values of 11.4 and 39.0 µM, respectively. Doxorubicin is known for a broad spectrum of antitumor activity [21].

EC_50_ values for compound **2** against both these cell lines were estimated to be above 50 µM, with cells observed to be fully viable at the tested concentration of 47.8 µM. Unfortunately, limited supply restricted investigations at higher concentrations. Like compound **1**, the EC_50_ values for compound **3** were above 100 µM for both PC3 and HT29, indicating moderate cytotoxicity by this compound against these two cell lines.

These initial, yet promising indications of cytotoxicity against two human carcinoma cell lines indicate the need for a full evaluation against a larger panel of cell lines, particularly for the two novel compounds.

## 3. Materials and Methods

### 3.1. General Experimental Procedures

Optical rotations were measured on an Anton Paar model MCP 300 polarimeter (Ostfildern scharnhausen, Germany) with a 0.5 dm cell. Nuclear magnetic resonance spectra were recorded on a Bruker Avance DRX-500 MHz spectrometer (Bruker BioSpin GmbH, Silberstreifen, Germany), equipped with a 5-mm broadband inverse probe and a 5-mm dual probe, employing deuterochloroform (CDCl_3_) as a solvent and referenced to tetramethysilane (TMS) as internal standard. ^13^C spectral editing were obtained by DEPT experiments. Chemical shifts (δ) were expressed in ppm and coupling constants in Hz. HR-ESI-MS spectra were taken on Bruker micro TOF focus II mass spectrometer (Bruker Daltonics GmbH, Bremen, Germany). Separation and purification were performed by column chromatography on silica gel 60 Å (230–400 mesh, SiliCycle Inc., Quebec City, QC, Canada). Compound detection in the thin-layer chromatography (TLC) plate was achieved by molybdenum spray detection. All the solvents were analytical grade.

### 3.2. Algal Sample Collection

Specimens of *C. cervicornis* (Dictyotaceae, Ochrophyta) were collected at Drunken Man’s Cay, Port Royal, Kingston, Jamaica in November 2015 at a depth of 15–20 m. Immediately after collection, the algal material was cleaned and air-dried for two days.

### 3.3. Extraction, Isolation, and Structural Elucidation of Compounds

The air-dried algal material (82.50 g) was extracted exhaustively at room temperature successively with hexane, methylene chloride, ethyl acetate, and methanol for three days each. After removal of the solvent, the methylene chloride extract yield was 2 g of a dark green gum residue. The methylene chloride extract was subjected to flash silica gel column chromatography (CC) (6 × 30 cm), eluting with 100% CH_2_Cl_2_, increasing portions of EtOAc in CH_2_Cl_2_ followed by increasing portions of methanol in EtOAc to produce 12 main fractions, which were monitored by thin layer chromatography (TLC).

Fraction 8 (100% CH_2_Cl_2_, 132 mg) was subjected to silica gel CC (1.5 × 8.5 cm), eluted with CH_2_Cl_2_ as solvent from which for 23 fractions, denoted F8.1 to F8.23 (5 mL each), were obtained. From these fractions, fraction 8.20 yielded compound **1** as a colorless gum (25.5 mg).

By crystallization in dichloromethane, fraction 11 yielded the pure compound (4*R*,9*S*,14*S*)-4,9,14-trihydroxydolast-1(15),7-diene 3 (53 mg) in the form of white orthorhombic crystals.

Fraction 12 (CH_2_Cl_2_/EtOAc (3:2), 102.7 mg) was subjected to silica gel CC (2.5 × 15.5 cm) eluted with Me_2_CO/CH_2_Cl_2_, 1:4 to afford 70 fractions (F12.1 to F12.70). From these fractions, fractions 12.15 to 12.17 afforded compound **2** (4 mg). All the isolated compounds were identified by the analysis of their 1D- and 2D-NMR spectra, together with EI-MS spectral analysis.

#### 3.3.1. 4*R*-Acetoxy-8*S*,9*S*-epoxy-14*S*-hydroxy-7-oxodolastane (**1**)

White gum; ${\left[\alpha \right]}_{D}^{25}$ +23° (*c* 0.005, CHCl_3_) ^1^H and ^13^C NMR data, see Table 1. (+)-HRESIMS *m*/*z*: 399.2029 [M + Na]^+^ (calculated for C_22_H_32_O_5_Na,399.2143).

#### 3.3.2. 4*R*-Hydroxy-8*S*,9*S*-epoxy-14*S*-hydroxy-7-oxodolastane (**2**)

White gum; ${\left[\alpha \right]}_{D}^{25}$ +18° (*c* 0.001, CHCl_3_) ^1^H and ^13^C NMR data, see Table 1. (+)-HRESIMS *m*/*z*: 357.2040 [M + Na]^+^ (calculated for C_20_H_30_O_4_Na, 357.1923).

#### 3.3.3. (4*R*,9*S*,14*S*)-4,9,14-Trihydroxydolast-1(15),7-diene (**3**)

White crystals; (+)-HRESIMS *m*/*z*: 343.2137 [M + Na]^+^(calculated for C_20_H_32_O_3_Na,343.2248). NMR data are consistent with literature values [8].

### 3.4. Cytotoxic Evaluation of Compounds

Human prostate carcinoma PC3 cells and human colon colorectal adenocarcinoma HT29 cells were obtained from ATCC (Manassas, VA, USA). PC3 was grown and maintained in F-12K medium supplemented with fetal bovine serum and penicillin/streptomycin in 5% CO_2_ at 37 °C, as recommended by ATCC. HT29 was cultured in McCoy’s 5A medium that was also supplemented with fetal bovine serum and penicillin/streptomycin in 5% CO_2_ at 37 °C.

When the cells reached 90–95% confluence, they were collected and transferred to 96-well plates at a concentration of 1.5 × 10^4^ cells per well. After 24 h of incubation, the cells were exposed to varying concentrations of the individual compounds for an other 24 h. After this time had elapsed, the cell viability was assessed using the MTS solution according to manufacturer’s instructions and as described elsewhere [15,16], using a µQuant Universal Microplate Spectrophotometer (Biotek Instruments Inc., Winooski, VT, USA) at 490 nm. The raw data was converted to percent cell viability in Microsoft Excel. (Redmond, WA, USA).

## 4. Conclusions

A chemical investigation of the organic extract of the Jamaican brown algae *Canistrocarpus cervicornis* (Dictyotaceae), collected off the coast of Port Royal, has led to the isolation of two new oxodolastanes (**1** and **2**) and the known dolastane (4*R*,9*S*,14*S*)-4,9,14-trihydroxydolast-1(15),7-diene (**3**) as a major diterpene constituent. Oxodolastanes **1** and **2** showed novelty in the C-4*R* acetoxy and hydroxyl group, respectively. To date, only three oxodolastanes have been discovered, including 10β-acetoxy-8α,9α-epoxy-14β-hydroxy-7-oxodolastane [3]. Structures and relative configurations were characterized by NMR spectroscopic techniques (1D and 2D). All the compounds were evaluated for their cytotoxicity. The results indicated that compounds **1** and **3** displayed moderate cytotoxicity for both PC3 and HT29 human cell lines. Thus, we suggest that oxodolastane diterpene compounds could play a role in drug discovery. However, a full evaluation against a larger panel of cell lines and new biological assays is needed.

## Figures and Tables

**Table 1:** ^1^H (and ^13^C) NMR data of compounds **1** and **2** determined at 500 (and 125) MHz in CDCl_3._

Position	1		2	
	δ_C_	δ_H_	δ_C_	δ_H_
1	149.6	-	149.6	-
2	26.3	2.66 (t, 12.2)	25.7	2.88 (d, 13.5)
		2.13 (m)		2.10 (m)
3	27.10	1.92 (d, 14.0)	28.5	1.78–1.82 (m)
		1.83 (m)		1.96 (t, 14.0)
4	81.7	4.81 (br s)	78.7	3.53 (br s)
5	43.7	-	41.2	-
6	50.7	3.77 (d, 15.1)	50.7	4.05 (d, 15.3)
		2.23 (15.1)		2.45 (d, 15.3)
7	207.7	-	207.8	-
8	72.0	-	70.7	-
9	82.0	-	80.7	-
10	21.7	1.81 (m)	21.8	1.82 (m)
11	36.7	1.31 (m)	36.8	1.21 (m)
12	40.7	-	40.8	-
13	41.2	1.92 (d,14.0)	42.6	2.24 (d,14.9)
		2.14 (m)		1.78 (m)
14	78.2	-	78.6	-
15	110.7	4.98 (br s)	109.3	4.91 (br s)
		5.01 (br s)		4.95 (br s)
16	19.5	1.07 (s)	18.3	0.96 (d, 6.5)
17	27.2	2.74 (sept.,6.6)	27.0	2.79 (sept., 6.6)
18	18.9	1.07 (s)	17.7	1.07 (s)
19	19.3	0.96 (d, 6.5)	18.1	0.96 (d, 6.5)
20	22.9	1.37 (s)	24.9	1.41 (s)
MeCO	169.0	-	-	-
MeCO	21.3	2.16 (s)	-	-
4-OH	-	-	-	3.49 (m)
14-OH	3.70 (s)	-	-	3.43 (s)

## Supplementary Materials

The following are available online at www.mdpi.com/1660-3397/15/6/150/s1. All 1D, 2D NMR, and HRESIMS spectra of compounds **1** and **2** are included.
