# Supplementary material for: Two New Oxodolastane Diterpenes from the Jamaican Macroalga Canistrocarpus cervicornis
Source: Mar Drugs. 2017 May 30;15(6):150. doi: 10.3390/md15060150 (PMC5484100; doi:10.3390/md15060150)
Supplement: Supplementary file 1 [file marinedrugs-15-00150-s001.pdf]

Supplementary Materials: Two New Oxodolastane Diterpenes from the Jamaican Macroalga *Canistrocarpus cervicornis*

Sanjay Campbell, JeAnn Murray, Rupika Delgoda and Winklet Gallimore

Index

Compounds Spectra

Figure S1. ^{1}H NMR spectrum of Compound **1** in $CDCl_3$ (500 MHz) S2

Figure S2. ^{13}C NMR spectrum of Compound **1** in $CDCl_3$ (125 MHz) S3

Figure S3. DEPT 90 NMR spectrum of Compound **1** in $CDCl_3$ (125 MHz) S4

Figure S4. DEPT 135 NMR spectrum of Compound **1** in $CDCl_3$ (125 MHz) S5

Figure S5. COSY spectrum of Compound **1** in $CDCl_3$ S6

Figure S6. HSQC spectrum of Compound **1** in $CDCl_3$ S7

Figure S7. HMBC spectrum of Compound **1** in $CDCl_3$ S8

Figure S8. NOESY spectrum of Compound **1** in $CDCl_3$ S9

Figure S9. HRESIMS spectrum of **1** S10

Figure S10. ^{1}H NMR spectrum of Compound **2** in $CDCl_3$ (500 MHz). S11

Figure S11. ^{13}C NMR spectrum of Compound **2** in $CDCl_3$ (125 MHz). S12

Figure S12. DEPT 90 NMR spectrum of Compound **2** in $CDCl_3$ (125 MHz). S13

Figure S13. DEPT 135 NMR spectrum of Compound **2** in $CDCl_3$ (125 MHz)... .... S14

Figure S14. COSY spectrum Compound **2** in $CDCl_3$. S15

Figure S15. HSQC spectrum Compound **2** in $CDCl_3$. S16

Figure S16. HMBC spectrum Compound **2** in $CDCl_3$. S17

Figure S17. NOESY spectrum Compound **2** in $CDCl_3$. S18

Figure S18. HRESIMS spectrum of **2**. S19

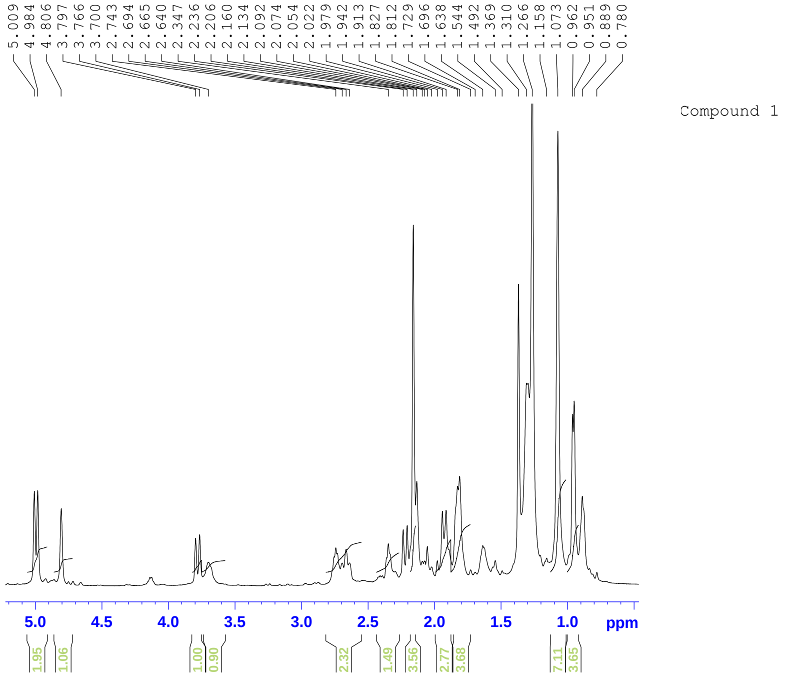

Figure S1. ^{1}H NMR spectrum of Compound **1** in $CDCl_3$ (500 MHz).

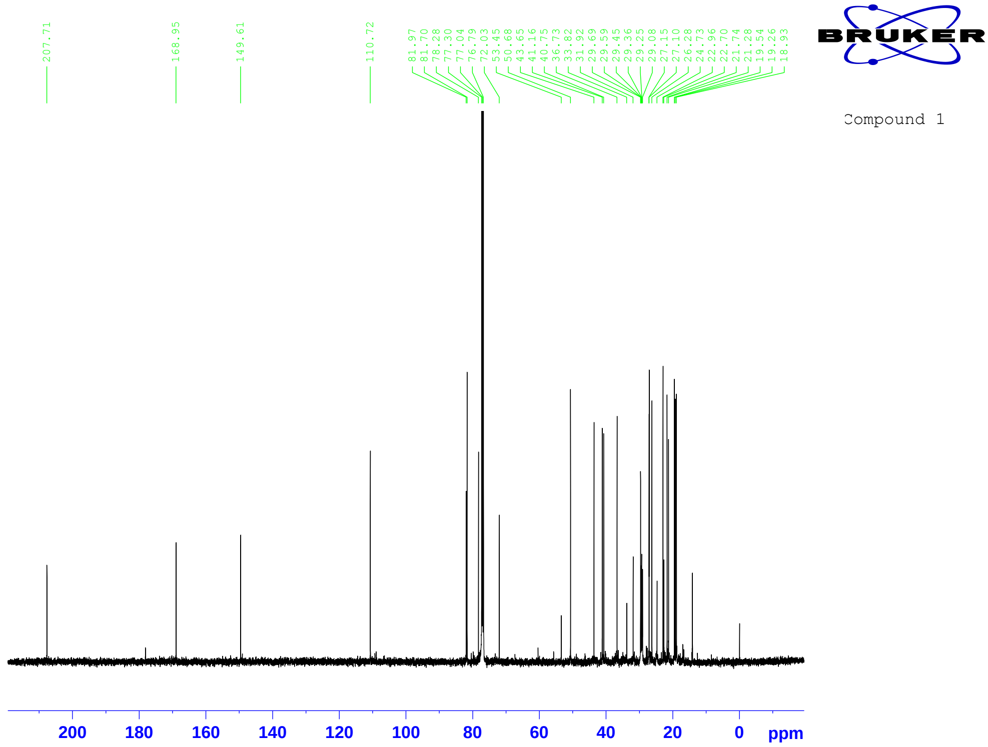

Figure S2. ^{13}C NMR spectrum of Compound **1** in $CDCl_3$ (125 MHz).

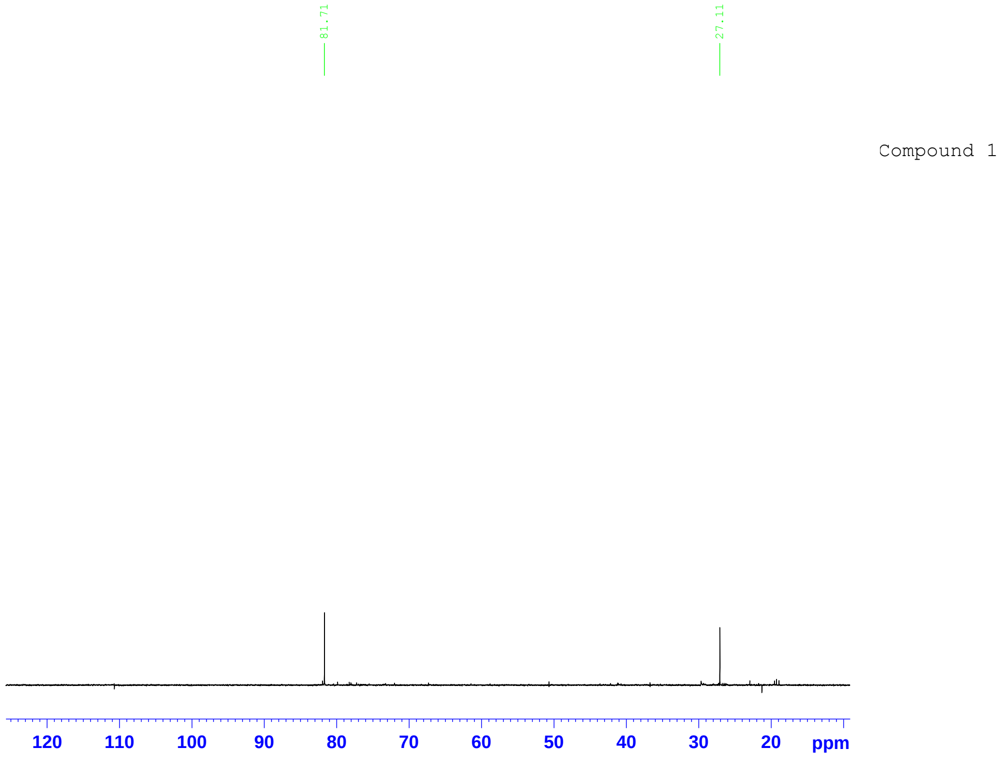

Figure S3. DEPT 90 NMR spectrum of Compound **1** in $CDCl_3$ (125 MHz).

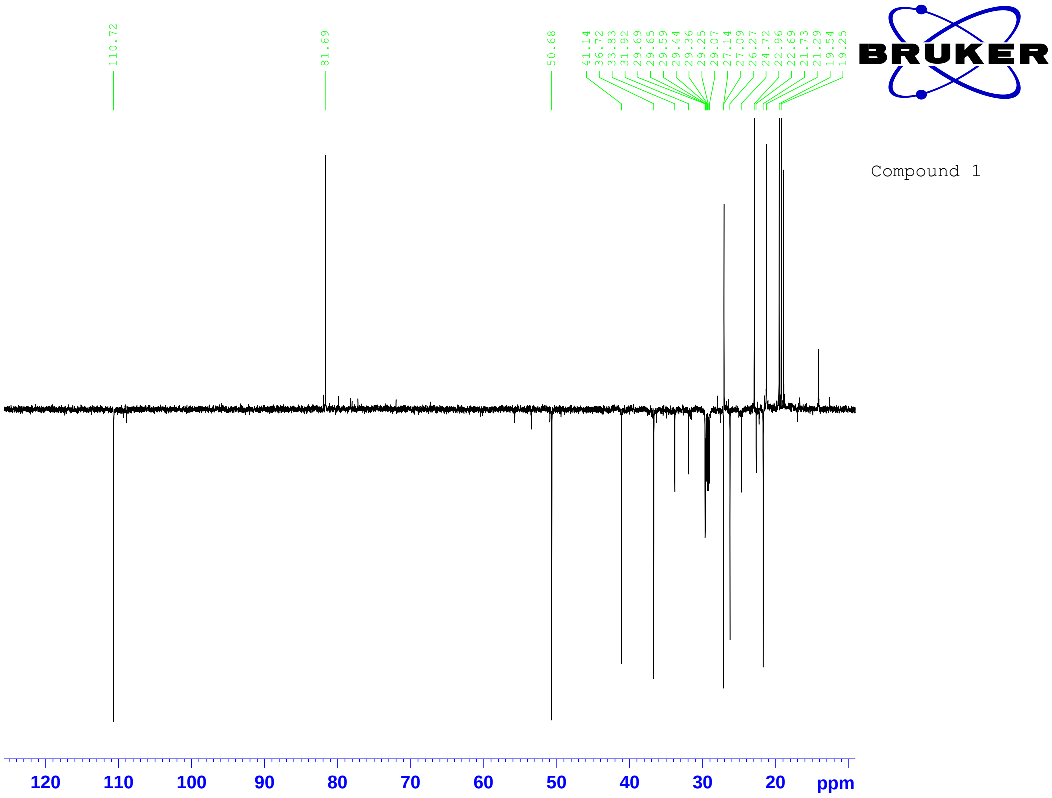

Figure S4. DEPT 135 NMR of spectrum of Compound **1** in $CDCl_3$ (125 MHz).

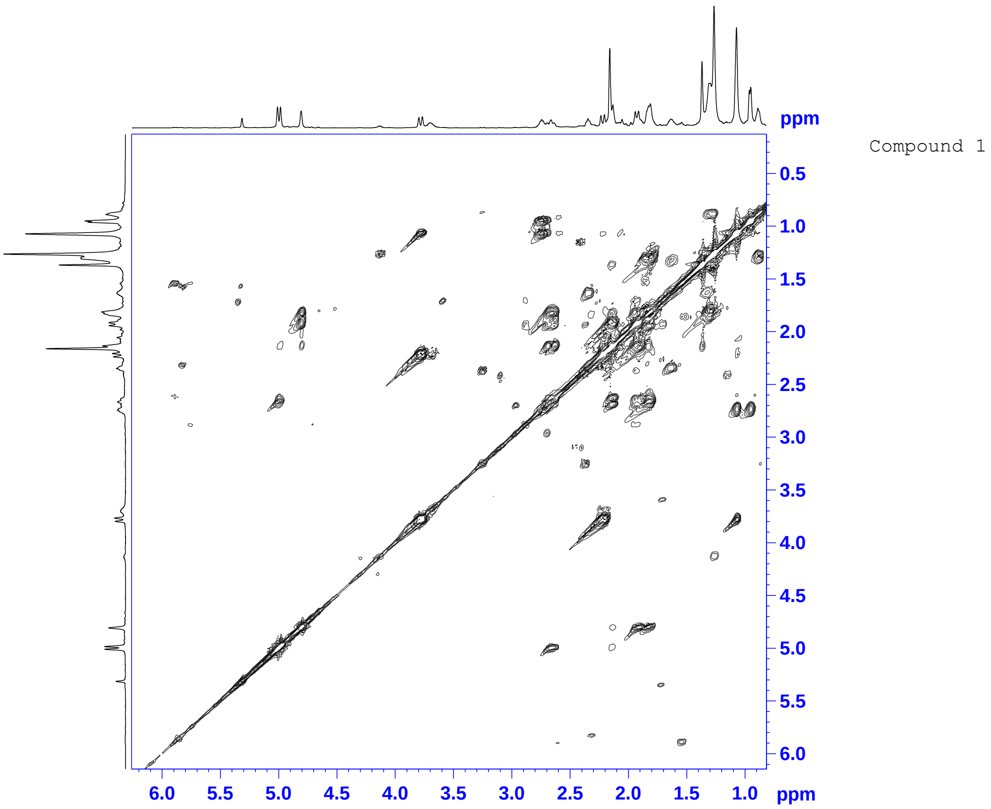

Figure S5. COSY spectrum Compound **1** in $CDCl_3$.

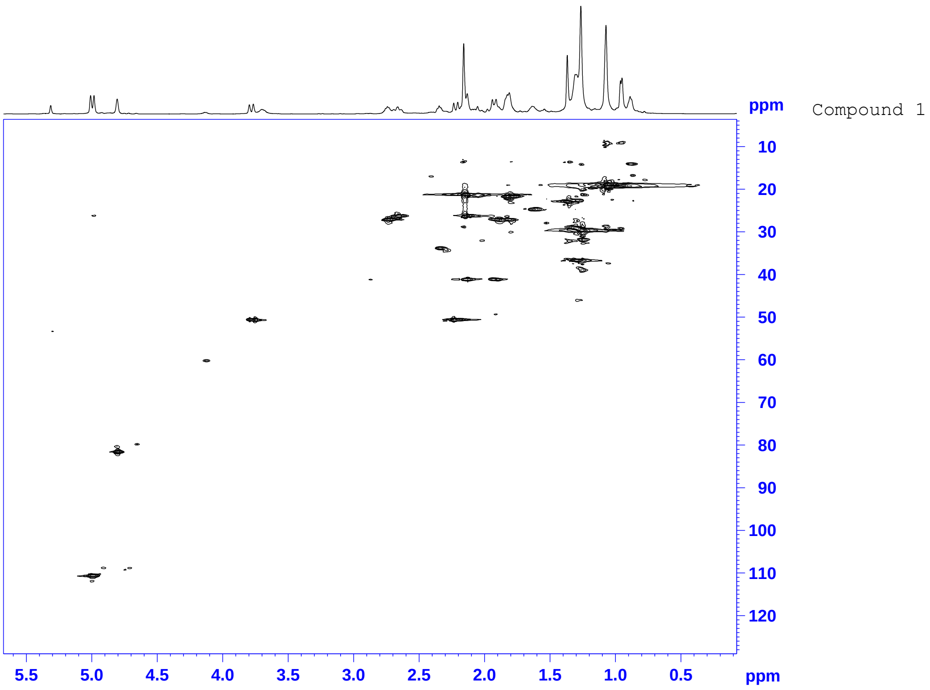

Figure S6. HSQC spectrum of Compound **1** in $CDCl_3$.

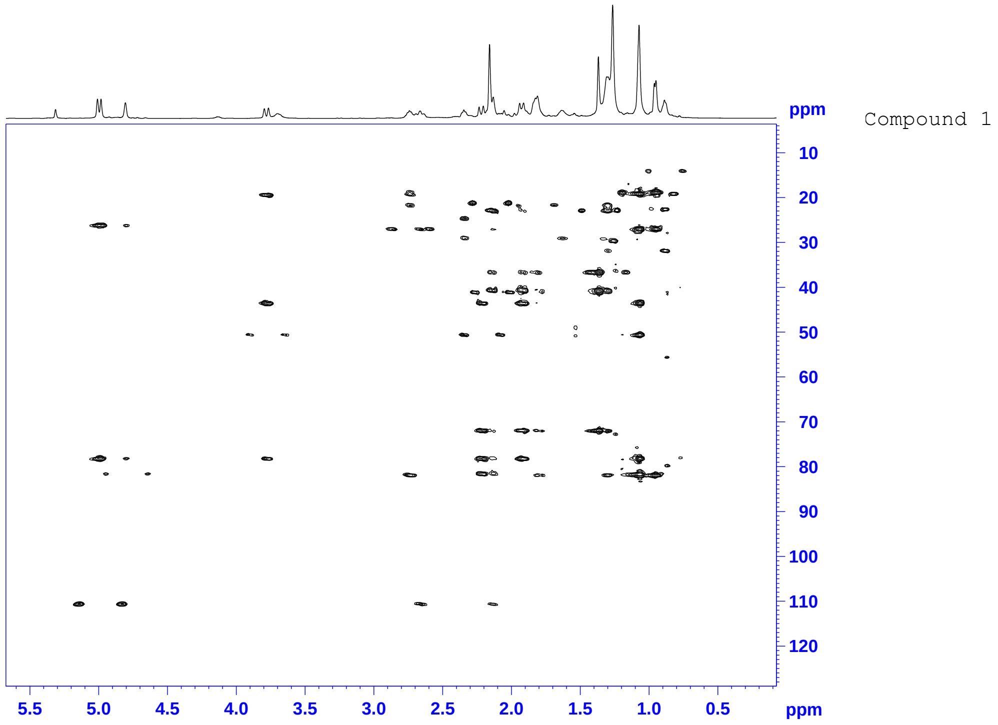

Figure S7. HMBC spectrum Compound **1** in $CDCl_3$.

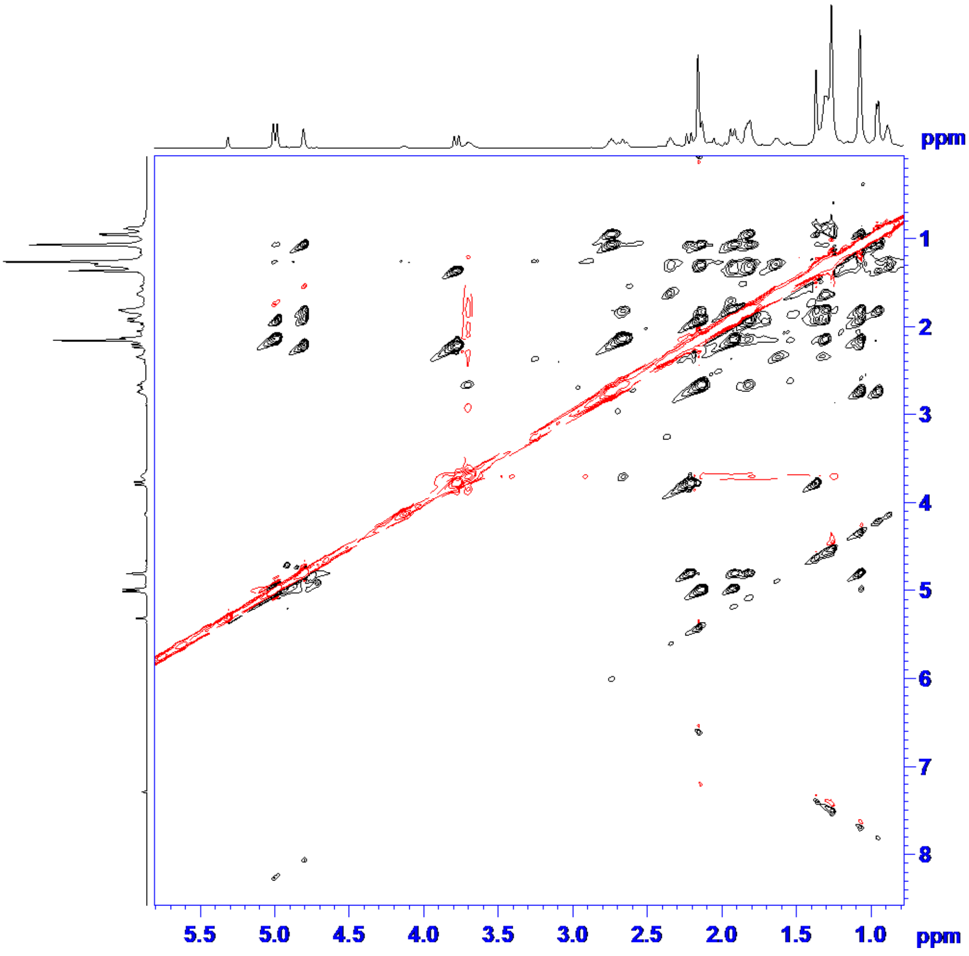

Figure S8. NOESY spectrum of Compound **1** in $CDCl_3$.

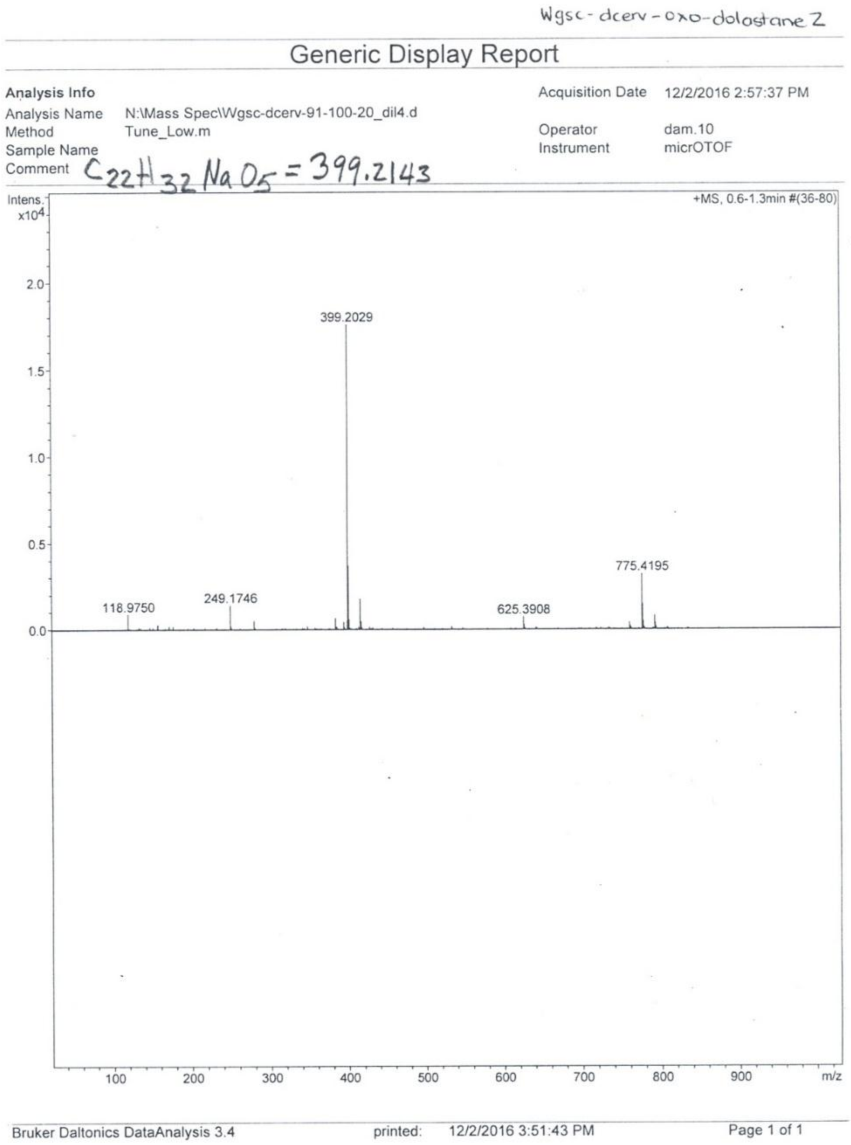

Figure S9. HRESIMS spectrum of Compound **1**.

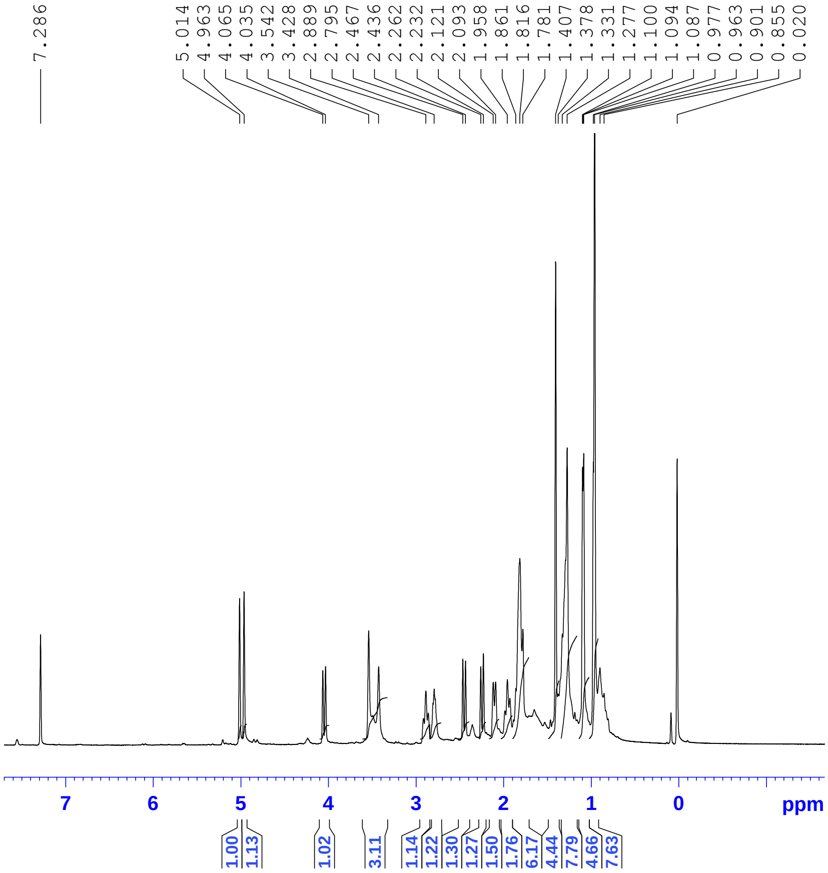

Figure S10. 1H NMR spectrum of Compound **2** in $CDCl_3$ (500 MHz).

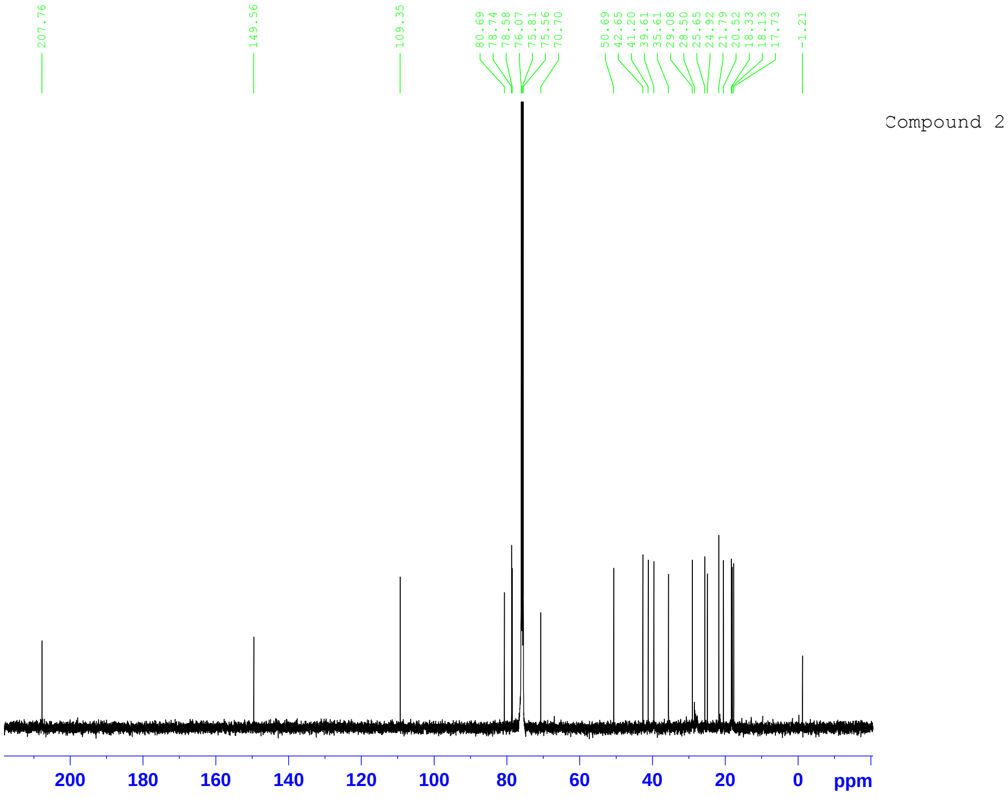

Figure S11. ^{13}C NMR spectrum of Compound **2** in $CDCl_3$ (125 MHz).

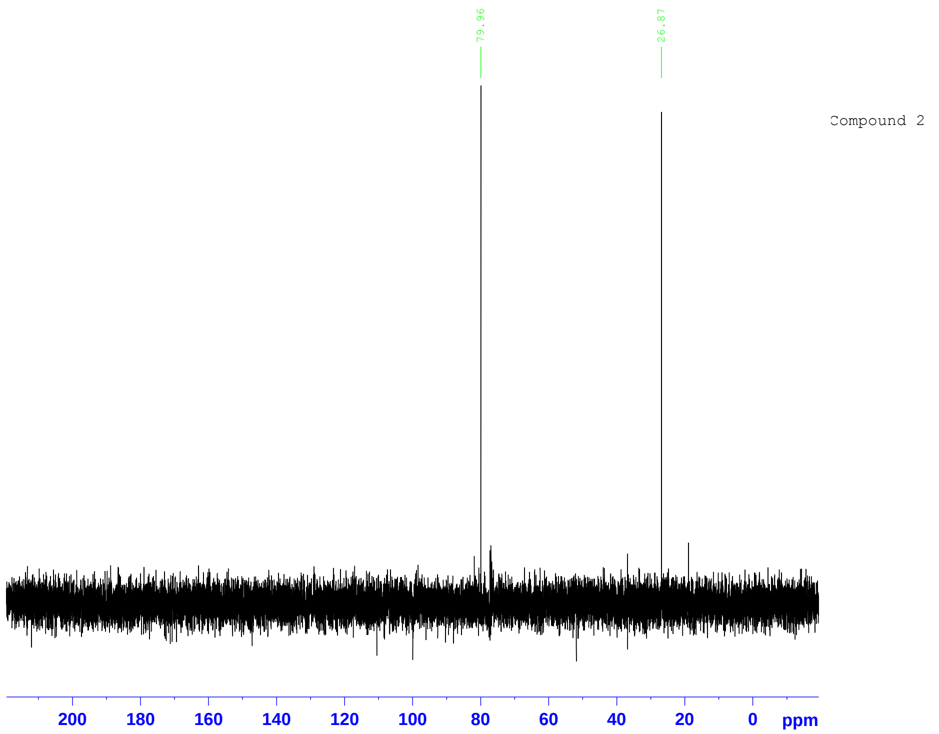

Figure S12. DEPT 90 NMR spectrum of Compound **2** in $CDCl_3$ (125 MHz).

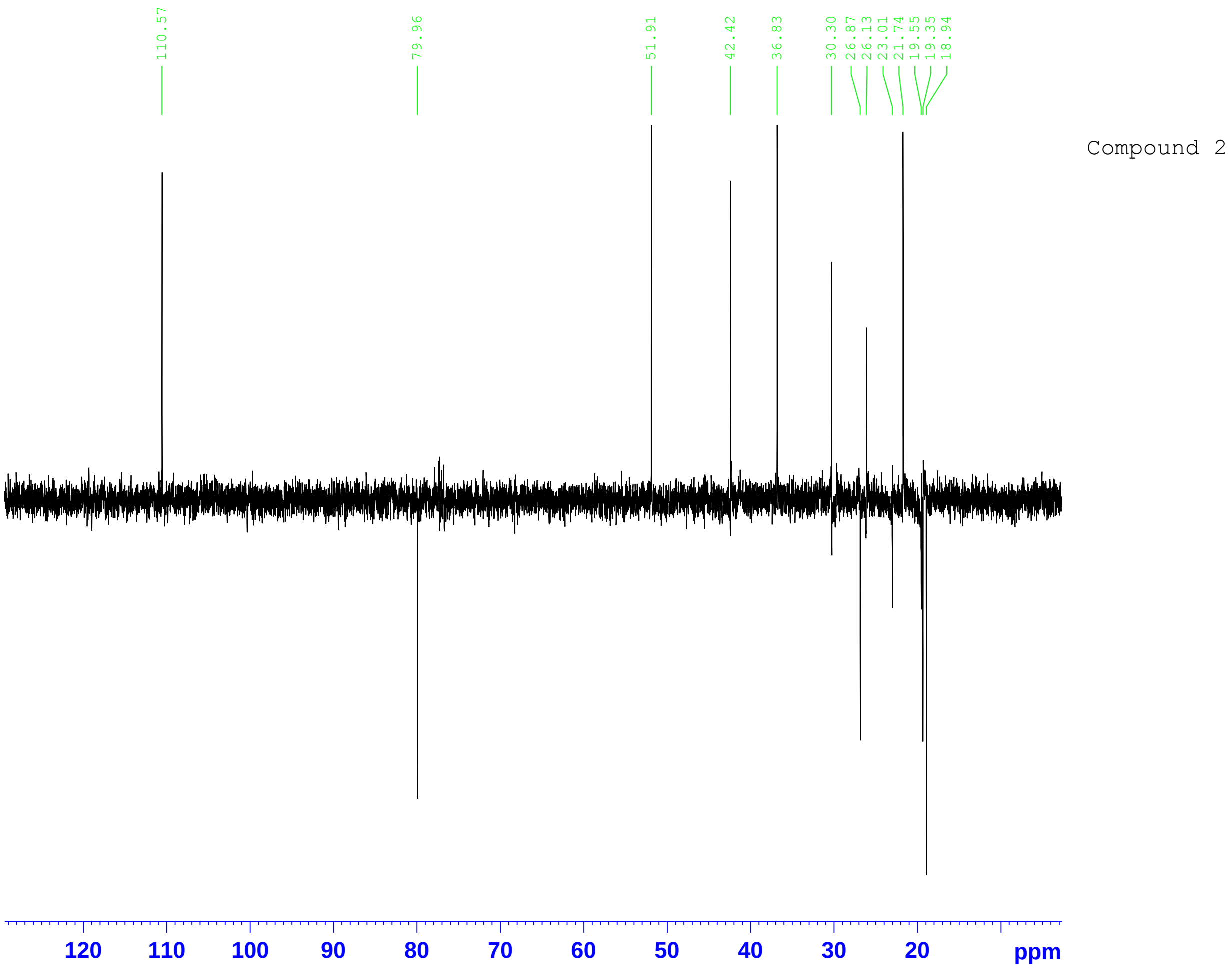

Figure S13. DEPT 135 NMR spectrum of Compound **2** in $CDCl_3$ (125 MHz).

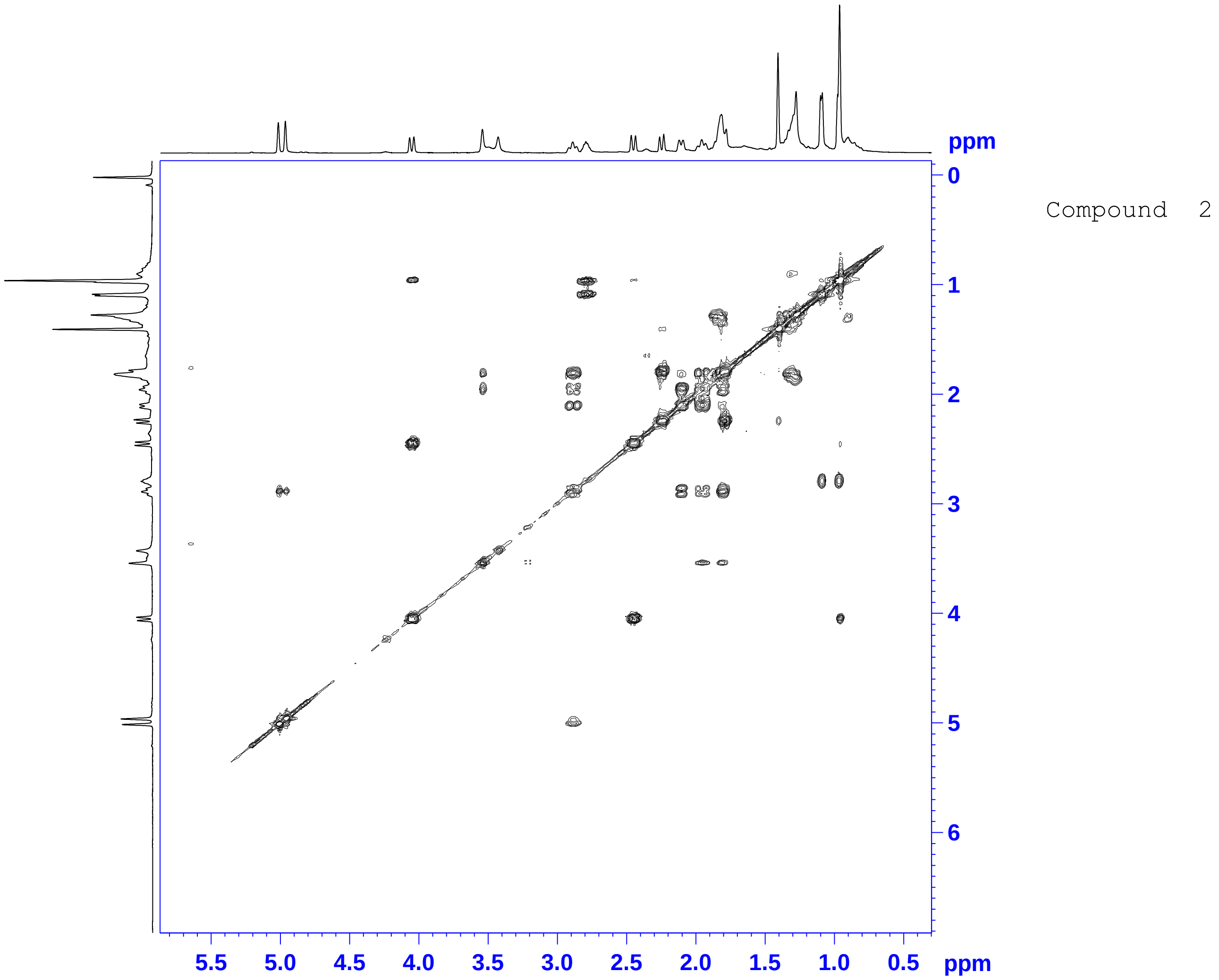

Figure S14. COSY spectrum Compound **2**.

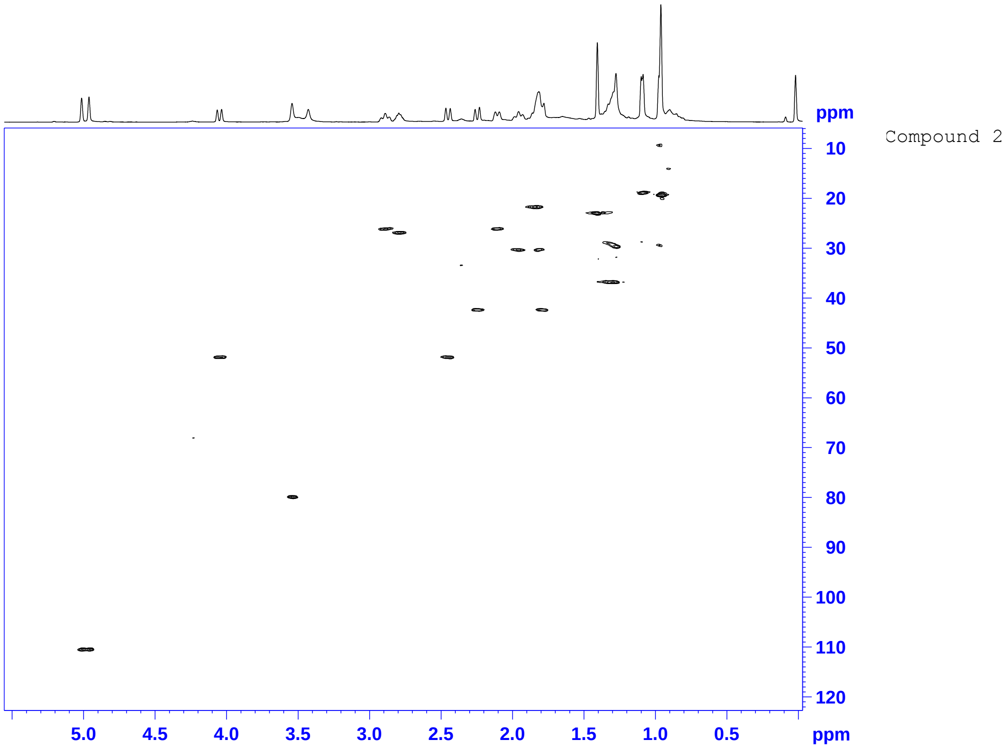

Figure S15. HSQC spectrum of Compound **2** in $CDCl_3$.

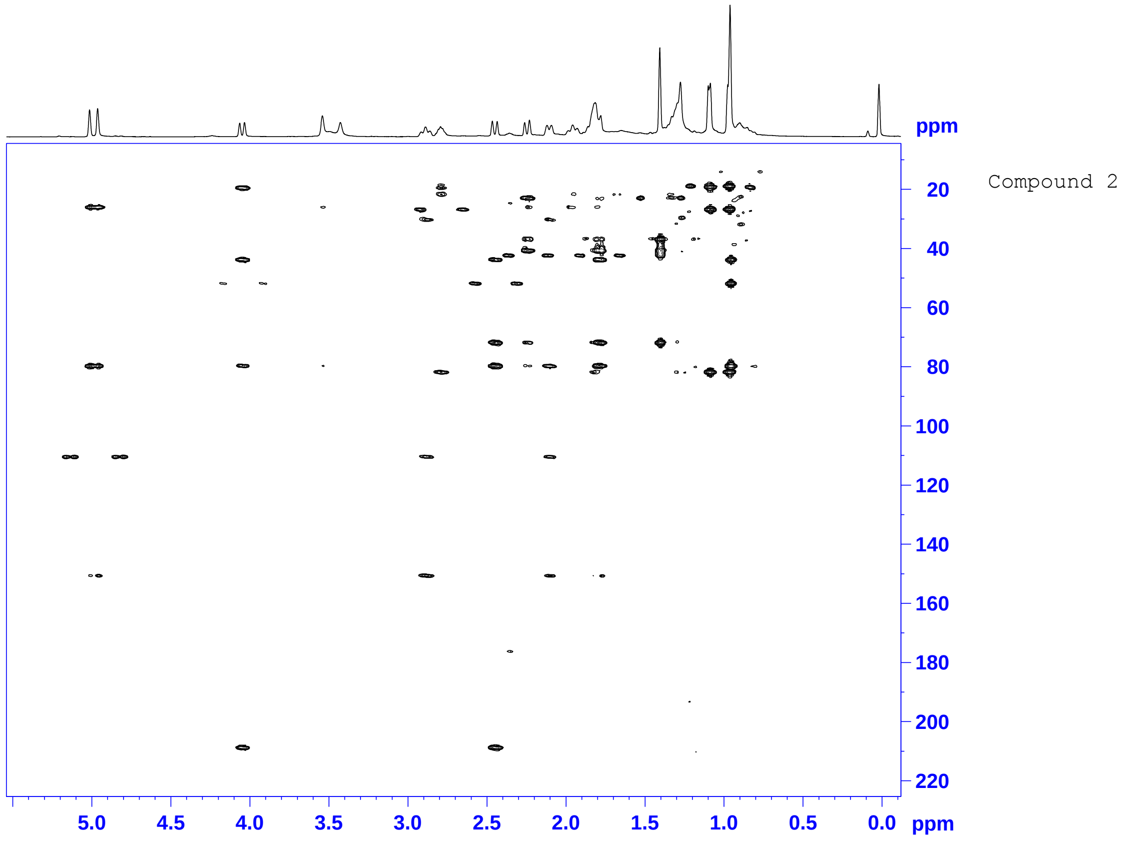

Figure S16. HMBC spectrum of Compound **2** in CDCl3.

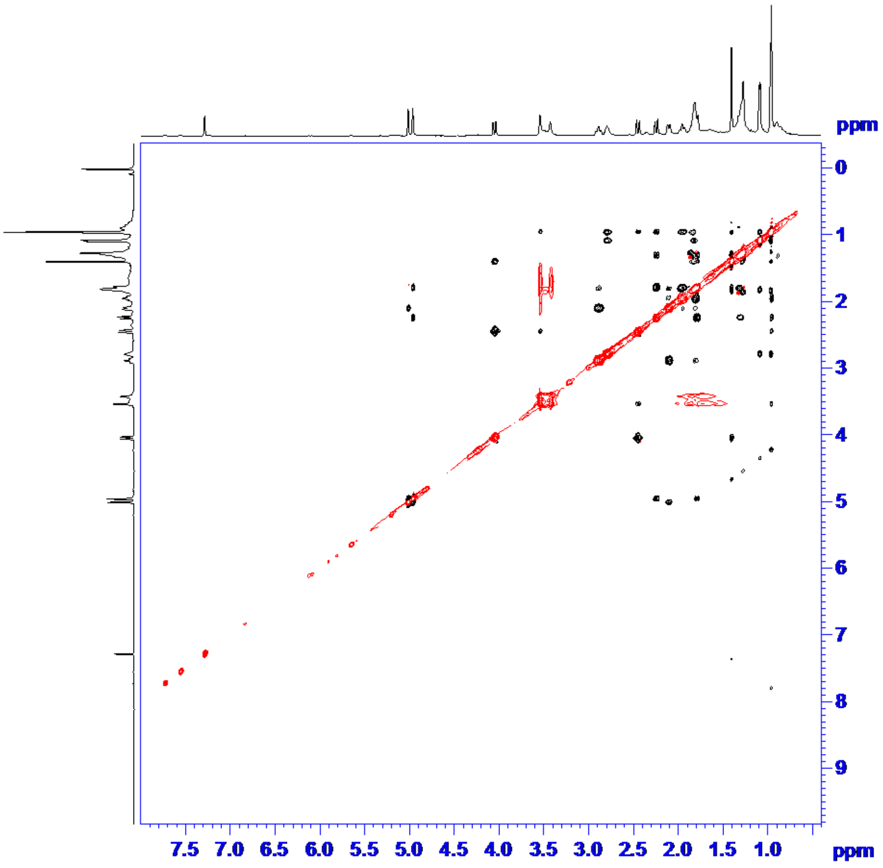

Figure S17. NOESY spectrum of Compound **2** in CDCl3.

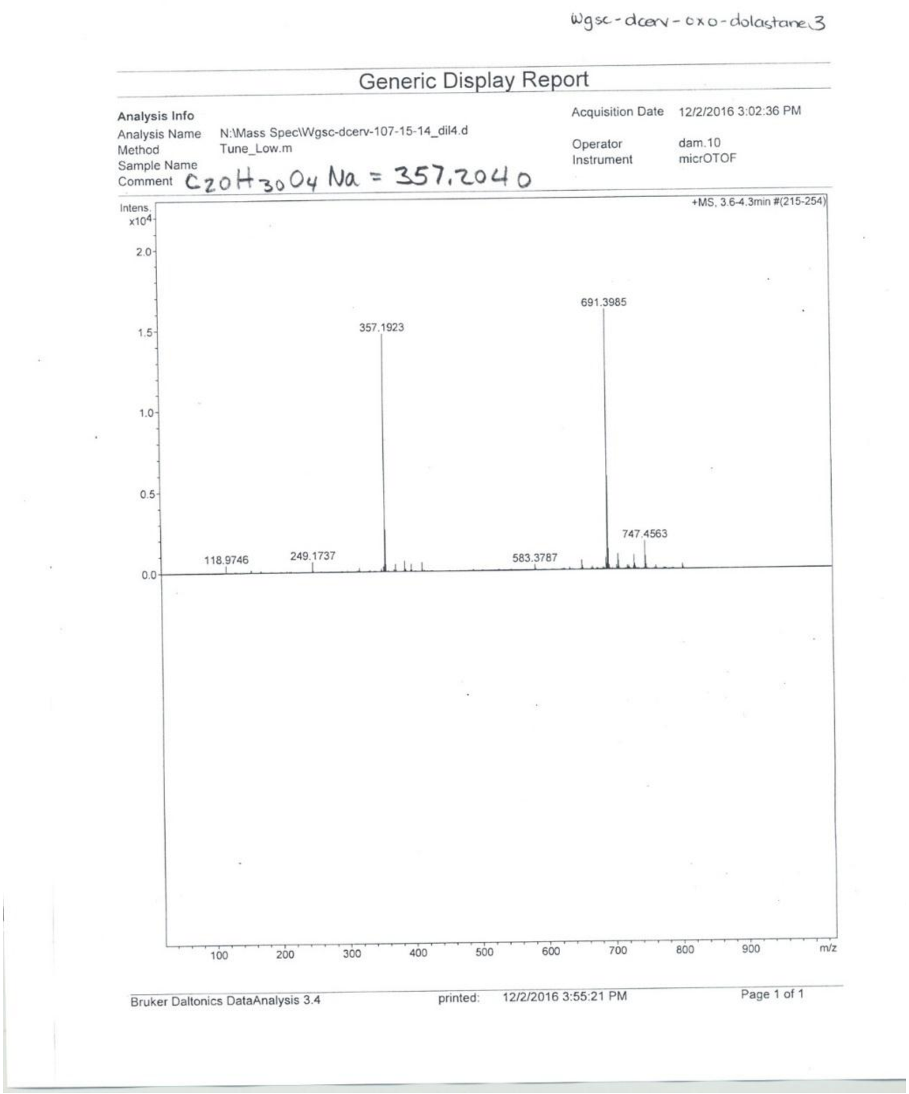

Figure S18. HRESIMS spectrum of **2**.
